# Dialysis: A Review of the Mechanisms Underlying Complications in the Management of Chronic Renal Failure

**DOI:** 10.7759/cureus.1603

**Published:** 2017-08-23

**Authors:** Sabitha Vadakedath, Venkataramana Kandi

**Affiliations:** 1 Biochemistry, Chalmeda Anand Rao Institute of Medical Sciences; 2 Department of Microbiology, Prathima Institute of Medical Sciences

**Keywords:** chronic renal failure, hypertension, dialysis, cardiovascular risk, glomerular filtration rate, end stage renal disease

## Abstract

Chronic renal failure (CRF) is the most prevalent, worldwide public health problem of the elderly population. The main cause of CRF is a damaged kidney. There are five stages of CRF based on the glomerular filtration rate (GFR), and stage 5 (GFR < 15 ml/min/1.73m^2^) is often called an end-stage renal disease (ESRD). In CRF, there is an accumulation of toxins and excess water due to compromised renal function. Dialysis is the preferred way to treat ESRD and remove accumulated toxins from the body. The cardiovascular risk associated with dialysis is 10 to 20 times higher in patients undergoing dialysis than in normal people. The inflamed kidneys and the process of dialysis also affect endothelial function, aggravating the risk of hypertension and cardiac problems. Therefore, both physicians and patients should be aware of the consequences of undergoing dialysis. There is an urgent need to educate CRF patients regarding facts about the disease, medications, dietary habits, and various measures required to manage the condition and lead a normal life. This paper attempts to delineate the mechanisms that could result in cardiovascular and other complications among CRF patients undergoing dialysis.

## Introduction and background

The process of removal of waste and extra water from blood is called dialysis [1]. It is an artificial replacement of kidney functioning, especially in renal failure cases. Dialysis cannot completely perform lost kidney function, but, to some extent, manages its activities by means of diffusion and ultrafiltration [2]. It is done in chronic renal failure (CRF) when the glomerular filtration rate falls below 15 ml/min/1.73m^2^ [3]. CRF is a condition where there is a loss of kidney function over a period of months or years. CRF can be diagnosed by measuring serum creatinine levels, which are a degradative product of muscle protein. Creatinine levels indicate the glomerular filtration rate (GFR) and in CRF, its activities are raised, indicating a lowered GFR [4]. There are five stages of CRF based on the GFR, and dialysis is preferred in stage 5 (GFR < 15 ml/min/1.73m^2^); this stage is also called end stage renal disease (ESRD) [5]. Dialysis is performed in CRF patients to remove accumulated toxins from the body. This procedure may be responsible for the development of oxidative stress, due to an imbalance between the overproduction of reactive oxygen species or toxins and a reduced defense mechanism of the body [4]. Oxidative stress disrupts the normal functioning of the cell. It was observed in a previous study that, in CRF, there could be raised plasma urate levels, further compromising the defensive mechanism of the body and increasing the oxidative stress [5].

The force with which blood flows through a blood vessel when the heart pumps blood is called blood pressure (BP), and it is measured with the help of a sphygmomanometer. In a normal, healthy person, the BP is 120/80 mmHg (systolic pressure (heart pumps)/diastolic pressure (heart relaxes)). If it is 140/90 mmHg, it is considered hypertension [6]. Hypertension increases the pressure of blood flow, which may damage blood vessels. In case renal blood vessels are involved, it leads to the accumulation of toxins and fluids, which further increases the blood pressure [7-8]. It is a known fact that hypertension alone is a risk factor for kidney diseases, and if it is associated with other complications, it leads to CRF.

The present paper elaborates the process of dialysis and how it influences the already nonfunctional kidneys (CRF). We also attempt to envisage the cardiovascular risk and metabolic abnormalities involved as a result of dialysis. The paper also focuses on the role of hypertension in kidney diseases and the associated cardiac risk among CRF patients.

## Review

The artificial process involving the removal of wastes and excess water from the blood is called dialysis. The criteria for undergoing dialysis is mainly disturbed renal functioning. Uremic syndrome, hyperkalemia, extracellular volume expansion, acidosis, not responding to medical therapy, creatinine clearance of 10 ml/min/1.73 m^2^, and bleeding diathesis (susceptibility to bleed due to coagulation defects) are the criteria for dialysis [9-11].

The renal functional capacity can be assessed by measuring serum creatinine/blood urea nitrogen (BUN) or by urea and creatinine clearance. There are two types of dialysis procedures; it may be hemodialysis (using a machine/artificial kidney-like apparatus) or peritoneal dialysis (using a peritoneal membrane as a filter). Peritoneal dialysis is recommended for younger patients because of its flexibility and can be performed at home. Hemodialysis is done for patients with no residual renal function. 

The mechanism of hemodialysis

In hemodialysis, the wastes and excess water are removed by using an external filter called a dialyzer, which contains a semipermeable membrane. The separation of wastes is done by creating a counter-current flow gradient, where blood flow is in one direction and the fluid of the dialyzer is in the opposite direction. Peritoneal dialysis uses the peritoneum as a natural semipermeable membrane and removes waste and water into the dialysate (the material or fluid that passes through the membrane of the dialysis).

**Figure 1:**
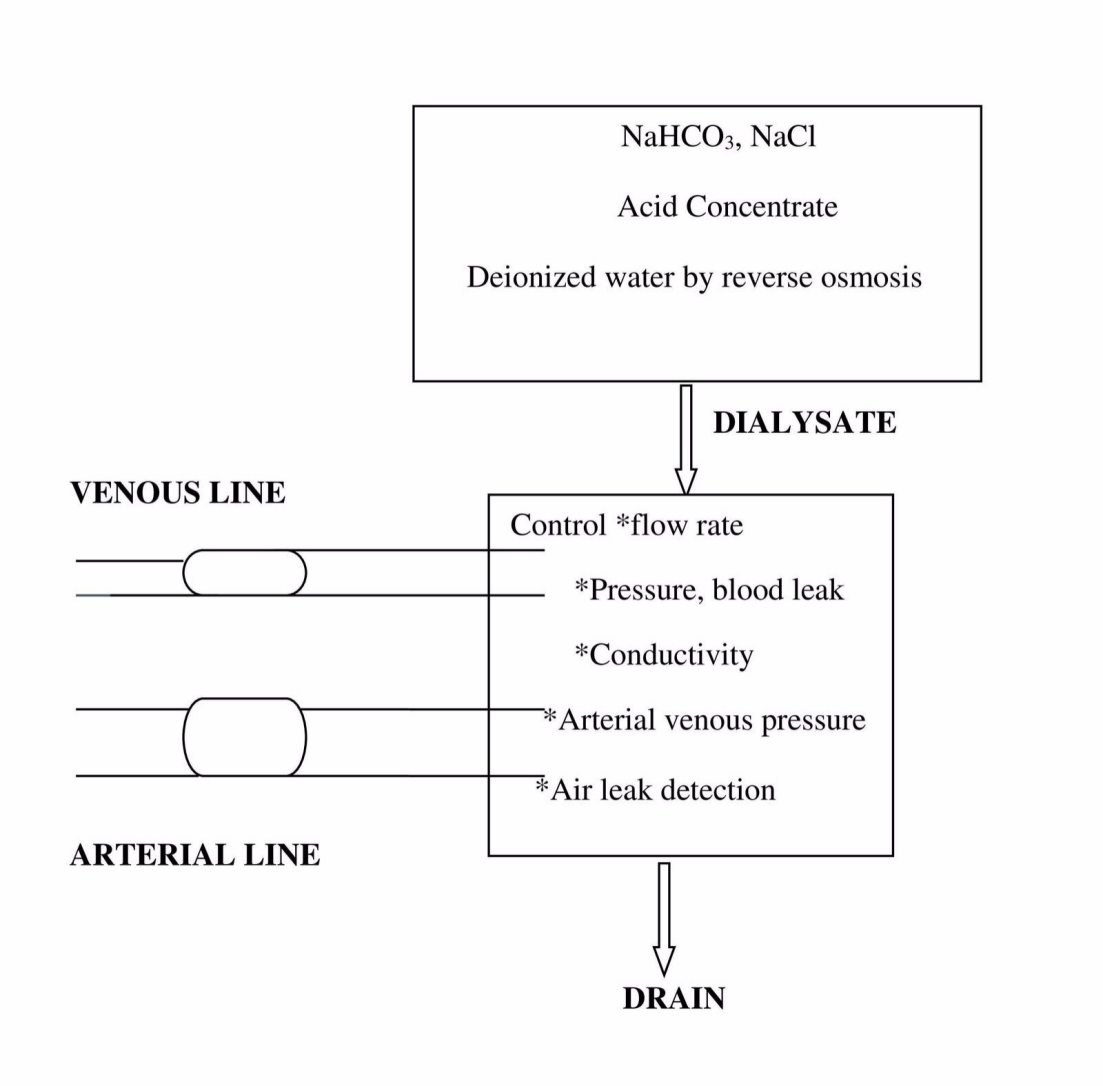
Diagrammatic Representation of a Dialyzer NaHCO_3_: sodium bicarbonate; NaCl: sodium chloride

The basic principle involved in dialysis is the movement or diffusion of solute particles across a semipermeable membrane (diffusion). Metabolic waste products, such as urea and creatinine, diffuse down the concentration gradient from the circulation into the dialysate (sodium bicarbonate (NaHCO_3_), sodium chloride (NaCl), acid concentrate, and deionized water). During their diffusion into the dialysate, the size of particles, in turn, determines the rate of diffusion across the membrane. The larger the size of the solute particle, the slower is the rate of diffusion across the membrane. Here, arteries carrying oxygenated blood from the heart are connected to a vein forming an arteriovenous shunt, which makes the vein strong (by forming muscles around it like an artery) enough to be punctured many times; its pressure is also monitored during the process of dialysis. The diagrammatic representation of a dialyzer is shown in Figure 1.

Cardiovascular complications and dialysis

**Figure 2:**
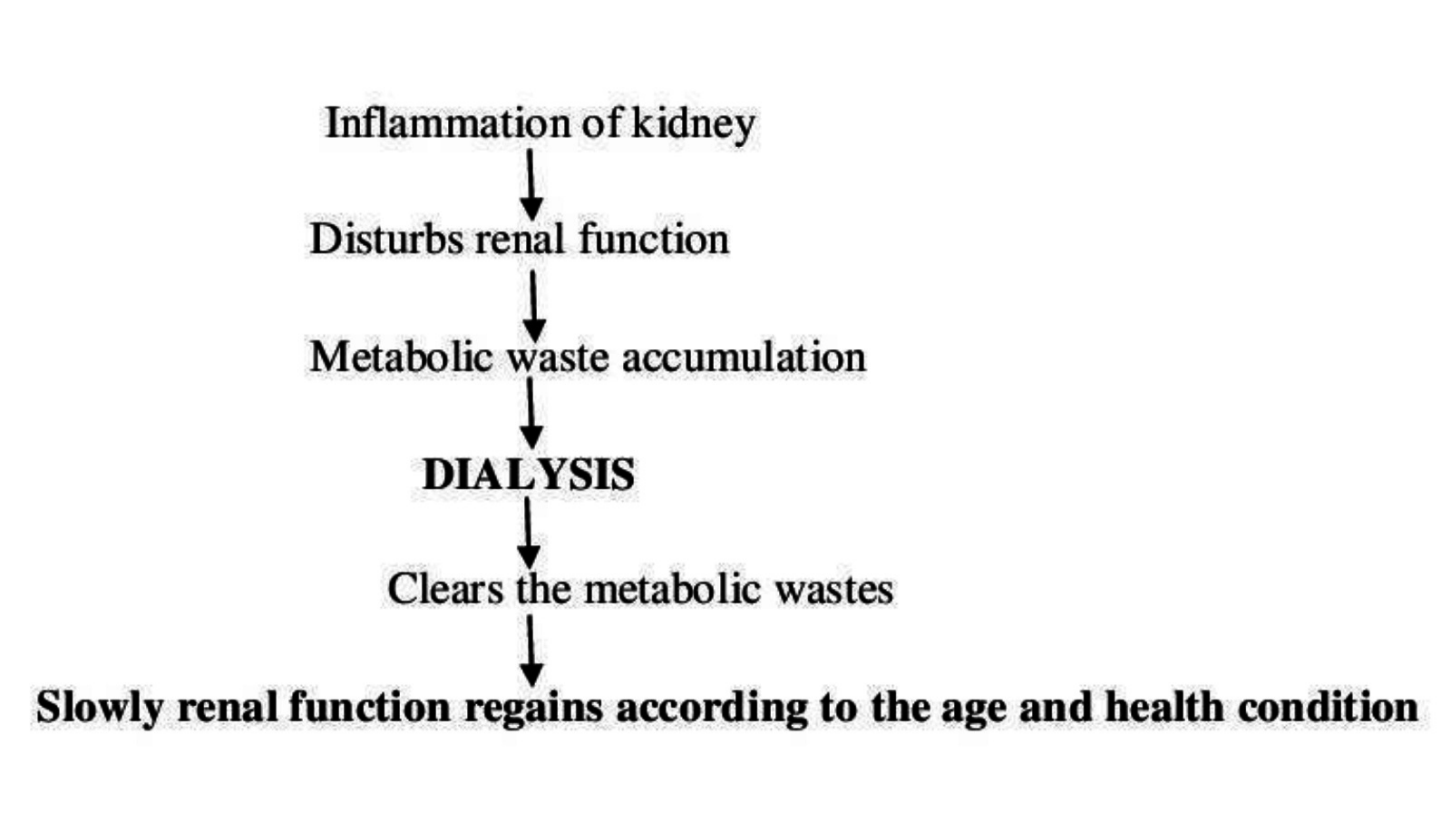
Flow Chart Showing the Stages and the Role of Dialysis

Dialysis could be associated with moderate (hypotension, muscle cramps, anaphylactic reactions) to severe (cardiovascular disease (CVD)) complications. Ongoing inflammation is the main reason for the diseased kidney, which does not respond to medications. Chronic inflammation disturbs the normal functioning of the kidneys, resulting in the accumulation of metabolic wastes in the body. The process of dialysis helps in the removal of toxins from the body and, slowly, the kidney may regain its function; this depends on the age and the health condition of the individual, as shown in Figure 2.

The presence of inflammation is an important factor in the development of oxidative stress in patients undergoing dialysis. During the process of dialysis, the membrane of dialysis is subjected to an immunological response by low molecular weight substances that include the IgG, the complement component, and makes this membrane permeable to granulocytes. The activated granulocytes in the blood stimulate the release of reactive oxygen species (ROS) and exaggerate the oxidative stress. It was also found that there are reduced trace elements, such as copper and zinc, and superoxide dismutase (SOD) levels among post-dialytic persons [12-13]. The nonfunctioning kidney activates macrophages, vascular cells, and various glomerular cells to produce free radicals, which further aggravate the oxidative stress, leading to a sequential change in organs, resulting in multiple organ failures and then death. Hypertension (uncontrolled due to inadequate treatment), hyperlipidemia, homocysteinemia, anemia, and the calcification of coronary arteries are the risk factors for CVD in dialysis patients. All these risk factors, alone or in combination, can alter cardiovascular dynamics [14-15].

Thyroid dysfunction and dialysis

**Figure 3:**
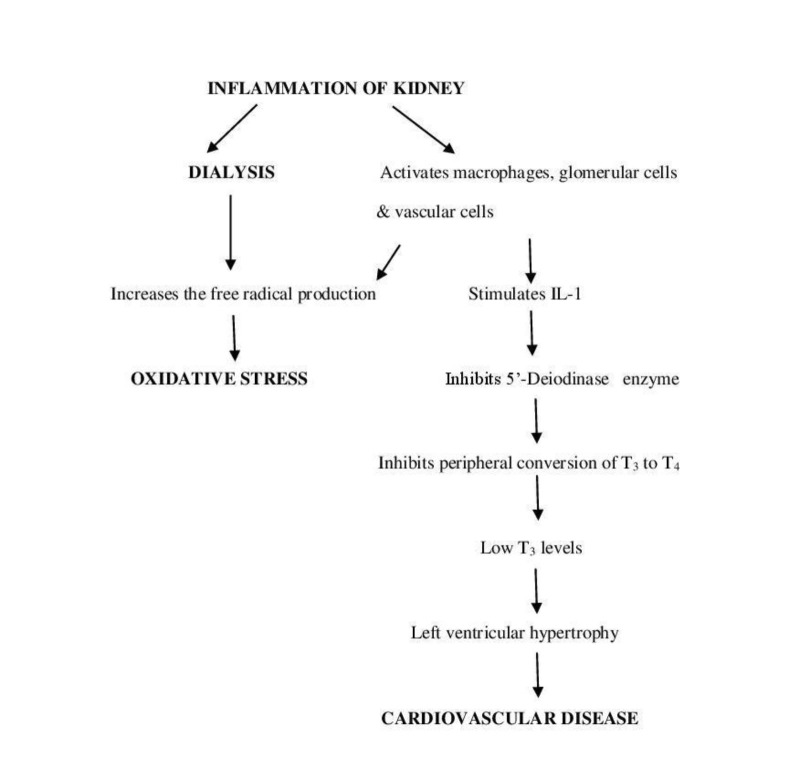
Flow Chart Showing the Consequences of Dialysis Associated with an Inflammation of the Kidney IL-1: interleukin 1; T_3_: triiodothyronine; T_4_: tetraiodothyronine/thyroxine

Thyroid hormones influence protein synthesis and cell growth, as evidenced by previous studies, which showed accelerated thyroid functioning during renal development in neonatal rats [16]. As a result, disorders of thyroid and kidney exist with a common etiological factor [17]. Thyroid function (low triiodothyronine (T_3_) levels) can be altered in dialysis, which may be attributed to the underlying cause—the inflammation. It was observed in experimental and clinical studies that interleukin signaling downregulates the peripheral conversion of tetraiodothyronine/thyroxine (T_4_) to T_3 _(Inhibition of 5'-deiodinase enzyme)*.* The low levels of T_3_ are associated with left ventricular hypertrophy and are considered as cardiovascular markers [18-20]. A flow chart demonstrating the side effects of inflammation associated with dialysis is shown in Figure 3.

Although dialysis is the preferred way to regain the functional ability of the kidneys, it could be responsible for certain side effects that include oxidative stress, thyroid disorders, and heart problems. Dialysis prolongs the life of an individual but cannot cure the underlying problem, so it is evident that the complications of dialysis could be due to the inflammation within the kidney.

Inflammation and dialysis

The inflammation of the kidneys may alter endothelial function, which could lead to decreased nitric oxide (NO) availability. The endothelial dysfunction can be predicted by the increased activities of asymmetric dimethyl arginine (ADMA). ADMA is an inhibitor of the enzyme "NO synthase," which is normally cleaved within the kidney [21]. Endothelial dysfunction also leads to proteinuria due to increased vascular permeability [22]. The improper functioning of kidneys disturbs several enzymes and receptors involved in lipoprotein metabolism (apo A1 (apolipoprotein A1)), particularly the high-density lipoproteins (HDL) and triglyceride-rich lipoproteins (chylomicrons, very low-density lipoproteins (VLDL), and low-density lipoproteins (LDL)) leading to hyperlipidemia [23]. It also causes the improper clearance of homocysteine, a sulfur-containing amino acid causing hyperhomocysteinemia and vitamin B_12_ deficiency anemia due to its influence on methionine synthase (an enzyme that helps to convert homocysteine to vitamin B_12_) [24].

**Figure 4:**
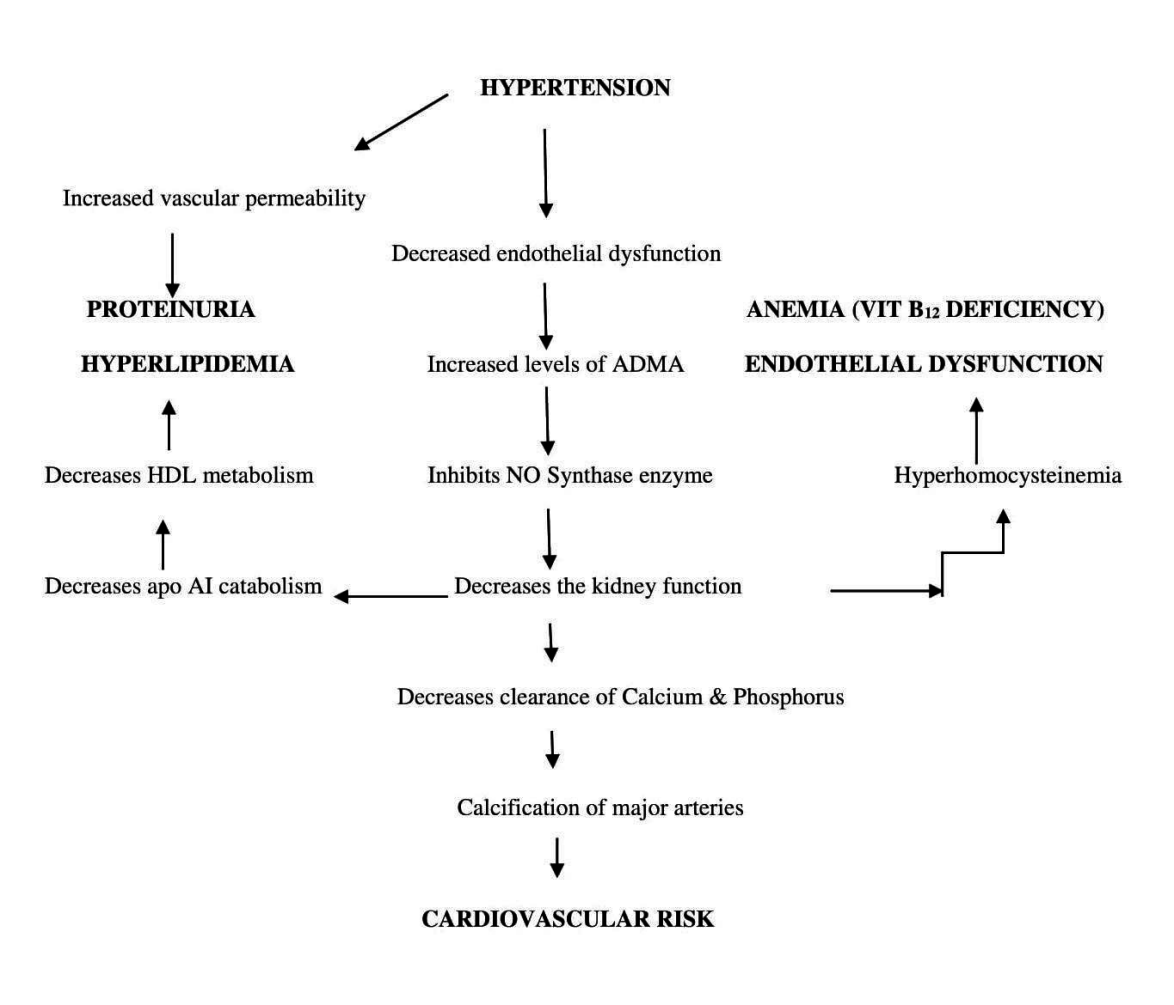
Flow Chart Showing Hypertension Related to Kidney Dysfunction and Its Effect on Various Metabolites ADMA: asymmetric dimethyl arginine; HDL: high-density lipoproteins; NO: nitric oxide; apo A1: apolipoprotein A1

Kidney dysfunction alters the lumen of blood vessels by inhibiting the cross-linking of collagen, making them atherogenic (narrows the lumen of the vessels) [25]. The kidney dysfunction may also affect the clearance of calcium and phosphorus, which could be responsible for the calcification of major arteries such as coronary arteries [26]. The calcification of major arteries can be assessed by measuring a glycoprotein, osteoprotegerin (OPG) [27]. The mechanism underlying kidney dysfunction and its effect on blood pressure and other metabolites is shown in Figure 4. 

In patients undergoing dialysis, there is an increased probability that the inflammation of the kidneys is accelerated, leading to further complications. Although the causes of inflammation are multifactorial, as discussed earlier, they also depend greatly on the membrane biocompatibility and the quality of dialysate. During dialysis, there is a possibility of the retention of inflammatory markers, the development of oxidative imbalance, and the activation of the complement [28-29]. CRF patients undergoing hemodialysis are at increased risk of developing several conditions, which include anemia, bleeding disorders, infection, electrolyte abnormalities, and cardiovascular dysfunction [30-32].

## Conclusions

In CRF patients, dialysis is the best method to remove accumulated toxins from the body and improve the quality of life. But this process, by itself, may complicate the condition due to its side effects. Individuals suffering from CRF, who are on dialysis, could be at increased cardiovascular and metabolic risk. Nowadays, dialysis is vigorously used even for small, treatable issues of the kidney.Therefore, the consequences of undergoing dialysis should be made known to both the physicians and the patients. There is an urgent need to educate CRF patients about facts related to the disease, medications, dietary habits, and the various measures required to manage the condition and lead a productive life.
